# Molecular mapping of *restorer*-*of*-*fertility 2* gene identified from a sugar beet (*Beta vulgaris* L. ssp. *vulgaris*) homozygous for the non-restoring *restorer*-*of*-*fertility 1* allele

**DOI:** 10.1007/s00122-014-2398-4

**Published:** 2014-10-07

**Authors:** Yujiro Honma, Kazunori Taguchi, Hajime Hiyama, Rika Yui-Kurino, Tetsuo Mikami, Tomohiko Kubo

**Affiliations:** 1 Research Faculty of Agriculture, Hokkaido University, N-9, W-9, Kita-ku, Sapporo, 060-8589 Japan; 2 Hokkaido Agricultural Research Center (HARC), National Agriculture and Food Research Organization (NARO), Memuro, Hokkaido 082-0081 Japan

## Abstract

*****Key message***:**

**By genetically eliminating the major** ***restorer*** **-** ***of*** **-** ***fertility*** **gene (** ***Rf*** **), a weak** ***Rf*** **gene was unveiled. It is an allele of** ***Z*** **, long known as an elusive** ***Rf*** **gene in sugar beet.**

**Abstract:**

In the hybrid breeding of sugar beet, maintainer-genotype selection is a laborious process because of the dependence on test crossing, despite the very low occurrence of this genotype. Marker-assisted selection (MAS) of the maintainer genotype is highly desired by sugar beet breeders. The major *restorer*-*of*-*fertility* gene (*Rf*) was identified as *Rf1*, and its non-restoring allele (*rf1*) was discriminated at the DNA level; however, some of the *rf1rf1* selections retained an as yet unidentified *Rf*, another target locus for MAS. The objective of this study was to identify this *Rf*. An *rfrf1* plant was crossed to a cytoplasmic male-sterile sugar beet and then backcrossed to obtain progeny segregating the unidentified *Rf*. The progeny exhibited partial male-fertility restoration that was unstable in single plants. The segregation ratio of restored vs. non-restored plants suggested the involvement of a single *Rf* in this male-fertility restoration, designated as *Rf2*. We confirmed the feasibility of molecular tagging of *Rf2* by identifying four shared amplified fragment length polymorphism (AFLP) fragments specific to 17 restored plants. Bulked segregant analysis also was performed to screen the *Rf2*-linked AFLP markers, which were subsequently converted into 17 sequence-tagged site markers. All the markers, as well two additional chromosome-IV-assigned markers, were linked to each other to form a single linkage map, on which *Rf2* was located. Our data suggested that *Rf2* is likely an allele of *Z*, long known as an elusive *Rf* gene in sugar beet. We also discuss the importance of *Rf2* for sugar beet breeding.

**Electronic supplementary material:**

The online version of this article (doi:10.1007/s00122-014-2398-4) contains supplementary material, which is available to authorized users.

## Introduction

Cytoplasmic male sterility (CMS) in plants is a maternally inherited inability to produce functional pollen (Schnable and Wise 1998; Chase 2007). Use of CMS can provide a large number of seed parents without manual or chemical emasculation; therefore, the trait has been employed for hybrid seed production in many crop species (Schnable and Wise 1998; Wise and Pring 2002). Hybrid seed production using CMS involves three lines, namely the CMS line, a maintainer line and a restorer line (Chen and Liu 2014); however, the latter may be omitted if male fertility is unnecessary for the final yield (Budar et al. 2006). The interactions of cytoplasmic genes and nuclear genes are a prerequisite for these three lines. There needs to be a male sterility inducing cytoplasm (designated as S), a normal cytoplasm (non-male sterility inducing) (N), and alleles of a nuclear fertility-restorer gene (*Rf*) that suppresses the action of S (Schnable and Wise 1998). The genotypes of CMS lines, maintainer lines and restorer lines are designated as [S]*rfrf*, [N]*rfrf*, and [N or S]*RfRf*, respectively (Budar et al. 2006). Note that the maintainer line and restorer line are different in terms of genotype, but are indistinguishable at the phenotypic level because plants of both lines are male fertile. In other words, the presence/absence of *Rf* in a male-fertile plant is difficult to determine without a genetic marker.

Sugar beet breeding owes much to CMS because all current cultivars are hybrids produced using CMS (Bosemark 1993). Sugar beet CMS used for hybrid seed production was first discovered by (Owen 1942, 1945) and this CMS (the so-called Owen-CMS) remains the only practical CMS to date (Panella and Lewellen 2005; Bosemark 2006). Currently, a major problem in the hybrid breeding of sugar beet is the rarity of the maintainer genotype (less than 5 % on average) (Bosemark 2006). The maintainer genotype is identified by a test cross using an annual CMS tester, a procedure in which a plant genotype is considered to be maintainer only when all the progeny are fully male sterile (Bosemark 2006); thus, maintainer selection of sugar beet is far from efficient. As a means to increase the efficiency of maintainer selection, marker-assisted selection (MAS) appears to be a promising strategy. In this context, the identification and characterization of sugar beet *Rf*s are valuable research objectives for sugar beet breeding.

Identification of sugar beet *Rfs* is rather difficult because fertility restoration tends to be incomplete and the segregation ratio often deviates from those expected from simple genetic models (Owen 1945; Nagao and Kinoshita 1962; Theurer and Ryser 1969; Bliss and Gabelman 1965). Several genetic models explaining fertility restoration of sugar beet CMS have been proposed with different numbers of involved genes and actions. These models could be summarized as follows: there is a principal *Rf* that appears in almost all the investigations (Owen 1945; Hogaboam 1957; Nagao and Kinoshita 1962; Bliss and Gabelman 1965; Pillen et al. 1993). Owen (1945) first described this *Rf* and designated it as *X*. Besides *X*, there may be some minor *Rf*s, one of which was termed *Z* by (Owen 1945).

Progress toward the characterization of *X* has been made recently. The *X* gene is located on chromosome III (Pillen et al. 1993) [we follow Schondelmaier and Jung (1997) for chromosome numbering] and an allele of *X* was cloned as *Rf1*, whose gene product is a protein resembling the yeast mitochondrial metalloprotease OMA1 (Matsuhira et al. 2012). On the other hand, little was known about *Z*, because its small effect has made its genetic study difficult. In addition, some investigations reported that fertility restoration could be explained in the absence of such a minor gene (Savitsky 1963; Theurer 1971; Pillen et al. 1993). *Z* was thought to be on chromosome IV on the basis of the observed linkage between fertility restoration and monogerm seed ball, the latter of which is conditioned by the *M* locus on chromosome IV (Hogaboam 1957; Roundy and Theurer 1974; Schondelmaier and Jung 1997). Hjerdin-Panagopoulos et al. (2002) detected *Z* as two-linked quantitative trait loci (QTLs) for fertility restoration that were located on chromosome IV. The precise map position of *Z* is unknown.

Despite a lack of detailed knowledge about *Z*, MAS for the maintainer genotype was attempted on the basis of *Rf1* polymorphism (Moritani et al. 2013). DNA markers linked to one of the non-restoring alleles (i.e., *rf1*) were developed to test the feasibility of MAS for the maintainer genotype (Moritani et al. 2013). As a result, *rf1rf1*-based MAS could enrich the maintainer genotype: up to 83 % of the selections had the maintainer genotype in some populations (Moritani et al. 2013). However, none of the selected *rf1rf1* plants from other populations had the maintainer genotype (Moritani et al. 2013). Therefore, an as yet unidentified *Rf* reduced the maintainer-genotype frequency in the *rf1rf1*-selection.

It was not known whether this unidentified *Rf* was an allele of *Z*, whose impact on maintainer-genotype selection has not been elucidated. If this unidentified *Rf* is an allele of *Z*, it will become very clear that *Z* is not a minor *Rf* but an important locus for sugar beet breeding. To this end, we focused our analysis on the sugar beet line that is the most recalcitrant against *rf1rf1*-based MAS, ‘TA-36’ (Moritani et al. 2013). A plant derived from the *rf1rf1*-selections was used in this study. Here, we report the molecular mapping of the novel *Rf* that reduces the efficiency of maintainer-genotype selection. On the basis of its chromosome IV localization, this *Rf* is probably an allele of *Z*, which is now becoming a practical target locus for sugar beet breeding.

## Materials and methods

### Plant materials

All the sugar beet lines and populations used in this study were developed at the Hokkaido Agricultural Research Center (HARC), National Agricultural and Food Research Organization (NARO), in Japan. ‘TA-33BB-CMS’ is an annual tester line having Owen-CMS and is devoid of any *Rf*. ‘E60’ is a selection of ‘TA-36’, an introduced cultivar from Germany (Moritani et al. 2013). BC_1_F_1_ and BC_1_F_3_ were grown in a greenhouse (20 °C, 24 h day with incandescent light at night).

### Male-fertility phenotyping

Anthers were visually inspected and evaluated according to Moritani et al. (2013), (Table 1). Five male-fertile classes (N, P, S, G and W, from fully fertile to fully sterile) were indexed from 4 to 0, respectively (Table 1). The mean of the indices calculated from three flowers (on average) on different branches of a plant was used as the male-fertility value of the plant. Male-fertility phenotyping of the B_1_F_1_ and B_1_F_3_ was done in the winters of 2010 and 2011, respectively.

**Table 1:** Classification of male sterility in this study

Class	Character of anther			Male fertility index
	Color	Dehiscence	Pollen production	
N	Yellow	+	+	4
P	Yellow, sometimes orange	Both + and − are seen	±	3
S	Yellow, sometimes orange	−, rarely +	−	2
G	Light green	−	−	1
W	White or brown	−	−	0

Adopted from Moritani et al. (2013) with some modifications

### DNA isolation

The procedure of Doyle and Doyle (1990) was used to isolate total cellular DNA from fresh green leaves.

### Amplified fragment length polymorphism (AFLP) analysis

AFLP analysis was performed using an AFLP Core Reagent Kit (Invitrogen, Carlsbad, CA, USA). *Eco*RI and *Mse*I were selected as the restriction endonucleases. Adapter-ligated DNA was pre-amplified using Takara *Ex* Taq (Takara Bio, Ohtsu, Japan) using pairs of primers in which one of the four nucleotides at the 3′ terminus as a selective nucleotide. The PCR protocol was 20 cycles of 94 °C for 30 s, 56 °C for 1 min and 72 °C for 1 min. For selective amplification, pairs of primers having three selective nucleotides were used. The PCR protocol was 94 °C for 5 min; 13 cycles of 94 °C for 30 s, 65 °C (annealing temperature was decreased by 0.6 °C/cycle) for 30 s and 72 °C for 1 min; and 13 cycles of 94 °C for 30 s, 56 °C for 30 s and 72 °C for 1 min. The amplified products were electrophoresed in the high-efficiency genome scanning system (Kawasaki and Murakami 2000; Kikuchi et al. 2003) using a non-denaturing 14 % polyacrylamide gel and TBE buffer (89 mM Tris, 89 mM boric acid, 2 mM EDTA, pH 8.0). The gel was stained with SYBR Green I nucleic acid gel stain (Takara Bio) and a Typhoon Trio Variable Mode Imager (GE Healthcare, Little Chalfont, UK) scanned the stained gel.

### Unidirectional selective genotyping and bulked segregant analysis (BSA)

Unidirectional selective genotyping was performed using 17 partially fertile plants with male-fertility values ranging from 2.0 to 3.0. BSA was performed according to the method described by Michelmore et al. (1991). Ten restored individuals and 15 male-sterile individuals from BC_1_F_1_ were used, and groups of five pre-amplified DNAs were pooled to establish two restored bulks and three male-sterile bulks, respectively.

### Conversion of AFLP markers to sequence-tagged site (STS) markers

AFLP bands were excised from the gel and the DNA fragments were eluted into TE buffer by repetitive freeze–thaw cycles. DNA fragments were then re-amplified using Green Go Taq Master Mix (Promega, Madison, WI) with its cognate selective primers for AFLP. The PCR products were electrophoresed through a 2 % agarose gel and purified using an Ultra Clean 15 DNA Purification Kit (MoBio Laboratories, Carlsbad, CA, USA). Purified PCR products were cloned into the pBluescript (SK+) vector using Ligation high ver. 2 (Toyobo, Osaka, Japan) and sequenced on an ABI3130 Genetic Analyzer (Applied Biosystems, Foster City, CA, USA) using a BigDye Terminator v3.1 Cycle Sequencing Kit (Applied Biosystems). Sequencher (Hitachi Software Engineering, Tokyo, Japan) was used for sequence analysis. STS markers were amplified using Green Go Taq Master Mix (the nucleotide sequences of the primers are shown in Table S1). The PCR protocols were 94 °C for 3 min; and 35 cycles of 94 °C for 30 s, 54 °C for 30 s and 72 °C for 1 min. For cleaved amplified polymorphic sequence (CAPS) analysis, amplified products were digested with restriction enzymes purchased from Takara Bio or New England Biolabs (Ipswich, MA, USA) and electrophoresed in a 2 % agarose gel or 10 % polyacrylamide gel.

### Statistical analysis, linkage analysis and quantitative trait locus (QTL) analysis

A Chi square test was done using R version 2.14.0 (R Development Core Team 2011). Fisher’s exact test was performed at the website of Gunma University, Japan (http://aoki2.si.gunma-u.ac.jp/exact/fisher/getpar.html) (accessed on 5 June, 2014). A linkage map was constructed using MAPMAKER/EXP ver3.0 (Lander et al. 1987). The map distances in centimorgans (cM) were calculated from recombination frequencies using the Kosambi function (Kosambi 1944). QTL analysis was performed using the simple interval mapping (SIM) method with MAPMAKER/QTL ver1.1b (Lincoln et al. 1993) and the composite interval mapping (CIM) method with WinQTL Cartographer ver2.5 (Wang et al. 2007), in which the logarithm of odds (LOD) threshold (*p* = 0.05) was generated by 1,000 permutation tests. CIM was performed using Model 6 at a walk speed of 1.0 cM and a window size of 10.0 cM. The confidence interval (CI) was defined as the region outside of which the log-likelihood fell by 1.0.

## Results

### Segregation of male fertility in the progeny of ‘E60’

We crossed ‘E60’ (a selection of ‘TA-36’, *rf1rf1* genotype) with ‘TA-33BB-CMS’ (seed parent) to obtain fertility restored F_1_ plants. One of the F_1_ plants was used as a pollen parent for backcrossing to ‘TA-33BB-CMS’, and the BC_1_F_1_ was obtained (115 plants). In the BC_1_F_1_, we noticed that male fertility often differed between flowers on a single plant. Hence, we first indexed the male-fertility phenotype as shown in Table 1, in which male fertility decreases as the value decreases (from 4 to 0). Subsequently, three flowers (on average) borne on different branches were evaluated for each of the 114 BC_1_F_1_ plants (one plant died before phenotyping) to calculate the plant’s mean index of male fertility (male-fertility value). The obtained male-fertility value distribution is shown in Table 2.

**Table 2:**
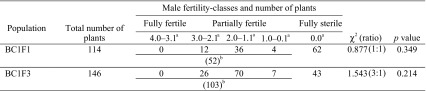
Segregation of observed male fertility

^a^Male-fertility value classes

^b^Sum of fertility restored plants

No plant was classified into the 4.0–3.1 class, which can be considered as fully fertile. On the other hand, we never observed any fertility restoration of ‘TA-33BB-CMS’ plants in our greenhouse. Thus we thought that the observed partial fertility (values 3.0–0.1) was conditioned by the *Rf* encoded in the genome of ‘E60’. Assuming a single dominant gene model for this partial fertility, 1:1 segregation of partially fertile plants and fully sterile plants could be expected. A Chi square test supported this genetic model (*p* = 0.349) (Table 2).

One of the BC_1_F_1_ plants with fairly high male fertility was self-pollinated to generate a BC_1_F_2_. The number of BC_1_F_2_ plants was insufficient for genetic analysis; therefore, one of the BC_1_F_2_ plants was self-pollinated to generate a BC_1_F_3_ (184 plants). We observed segregation of male fertility in 146 plants of this BC_1_F_3_ (38 died before phenotyping), and the male-fertility value of each plant was investigated (Table 2). As was the case with BC_1_F_1_, no fully fertile plant was observed. A single dominant gene model could also explain the occurrence of partially fertile plants (Chi square test for 3:1 segregation; *p* = 0.214) (Table 2). On the basis of these results, we concluded that ‘E60’ has an *Rf* that restores partial pollen fertility to an Owen-CMS plant. Hereafter, this *Rf* is designated as *Rf2*.

### Molecular markers linked to fertility restoration

BSA appeared to be an adequate method to obtain molecular markers tightly linked to *Rf2*; however, varying degrees of male fertility also suggested that this trait may be influenced by other minor gene(s) and/or environmental factors (i.e., it may be a quantitative trait), hence the feasibility of using BSA for this trait was uncertain. Therefore, before BSA, the presence of molecular markers associated with the observed fertility restoration needed to be confirmed.

We used a unidirectional selective genotyping approach (Foolad and Jones 1993; Navabi et al. 2009) in which AFLP fragments shared by 17 restored-BC_1_F_1_ plants (male-fertility values >2.0), but missing from ‘TA-33BB-CMS’, were sought. We tested 712 primer combinations that generated approximately 17,000 AFLP fragments, and found four fragments that appeared to be specific to the 17 restored plants (Table 3). The presence or absence of these four fragments was examined in 38 restored and 34 non-restored BC_1_F_1_ plants (Table 3). The distribution of the four bands was significantly biased toward fertility restored plants (Fisher’s exact test; *p* < 0.001), suggesting the feasibility of using BSA.

**Table 3:** Distribution of four AFLP markers in 72 plants

Pair of primers for selective amplification	Size of AFLP marker fragment (bp)	Presence/absence of the marker	Number of plants		Total number of plants	*p* value (2 × 2 contingency table, Fisher’s exact test)
			Fertility restored	Fully sterile		
E-CCC^a^/M-CCG^a^	290	+	31	0	72	6.46 × 10^−14^
		−	7	34		
		N/A^b^	0	0		
E-AAC^a^/M-CCG^a^	150	+	30	1	72	4.25 × 10^−12^
		−	7	33		
		N/A^b^	1	0		
E-AAC^a^/M-GAG^a^	120	+	32	0	72	1.05 × 10^−14^
		−	6	34		
		N/A^b^	0	0		
E-ACC^a^/M-CTG^a^	170	+	31	1	72	6.98 × 10^−13^
		−	6	33		
		N/A^b^	1	0		

^a^E- and M-denote *Eco*RI- and *Mse*I primers, respectively. The following three letters indicate selective nucleotides

^b^Not available

**Fig. 1:**
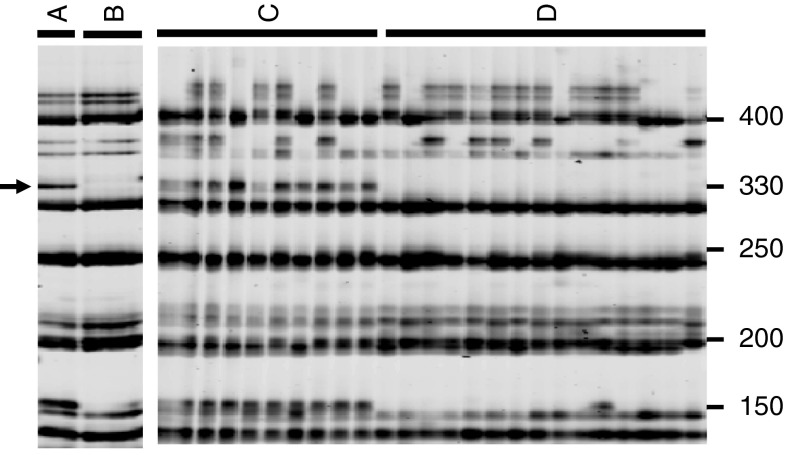
Images of the AFLP pattern using two restored bulks (lanes indicated by *A*), three non-restored bulks (*B*), ten fertility restored plants of BC_1_F_1_ (*C*), and 15 fully male-sterile plants of BC_1_F_1_ (*D*). Selective primers were *Eco*RI-CCC and *Mse*I-GAC. An *arrow* indicates an AFLP fragment associated with fertility restoration. *Size markers* are shown on the *right* (bp)

We conducted BSA using two restored bulks and three non-restored bulks made from the BC_1_F_1_. A total of 1,836 primer combinations were tested, and the number of AFLP fragments specific to the two restored bulks was 114. The presence or absence of these 114 fragments was examined in each of the bulked plants (Fig. 1). The number of AFLP fragments apparently associated with fertility restoration was 36; however, genetic mapping needs highly reproducible markers (i.e., STS markers) rather than AFLP fragments.

**Fig. 2:**
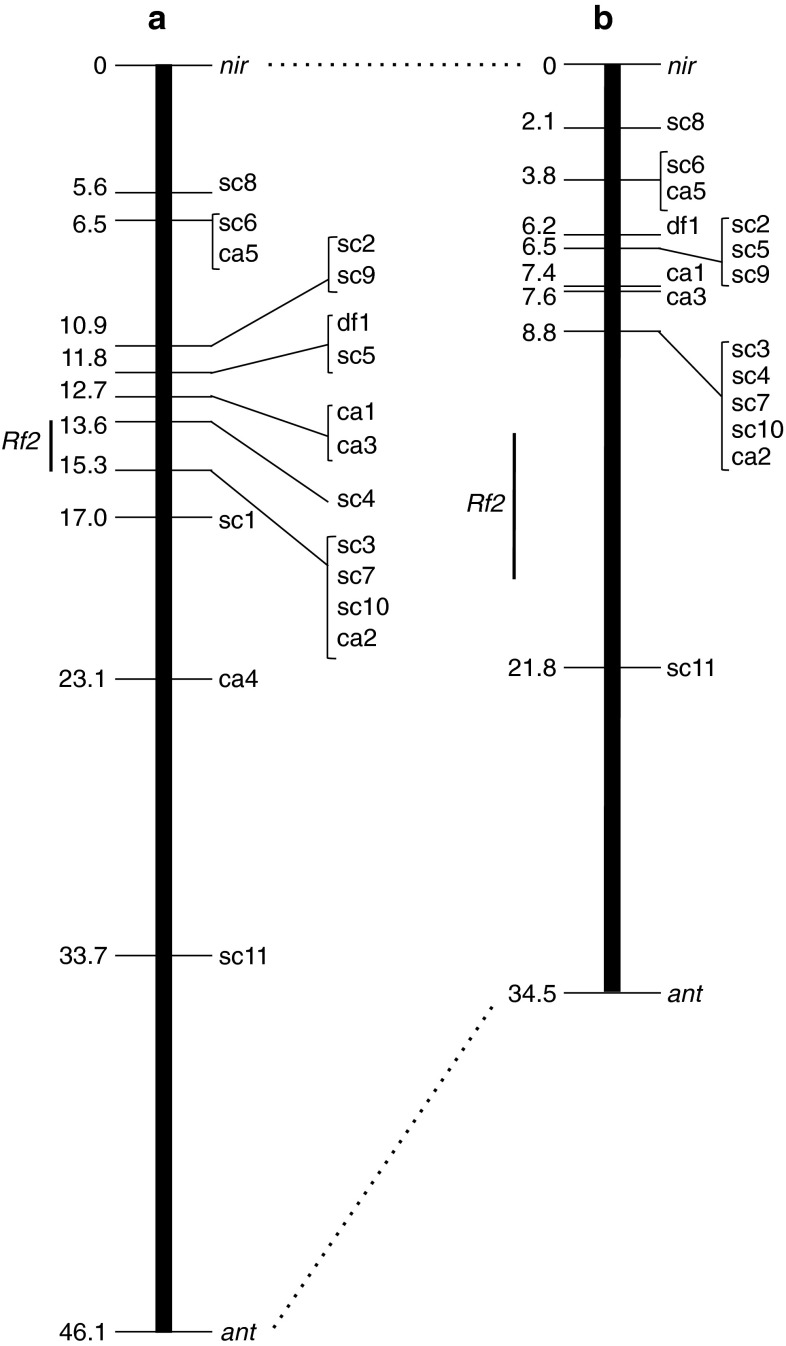
Linkage map of the chromosomal region around *Rf2* in BC_1_F_1_ (**a**) and BC_1_F_3_ (**b**) populations. Two chromosome-IV-assigned markers are connected by *dotted lines*. The confidence intervals of QTL for fertility restoration are indicated by *vertical bars* labeled with ‘*Rf2*’. Details of the *markers* are shown in Table S1. Map distances are shown in the *left* of each maps (cM). In the linkage map shown in **b**, repulsion of two regions (*nir*-ca2 and sc11-*ant*) was assumed in the linkage analysis

For accurate genetic analysis, we first conducted molecular cloning of the 36 AFLP fragments, from which we obtained 17 nucleotide sequences. Based on these sequences, we designed 17 pairs of PCR primers. Eleven of the 17 sequences were PCR amplified from restored plants, but not from non-restored plants of the BC_1_F_1_ [sequenced-characterized amplified region (SCAR) markers] (prefixed by ‘sc’ in Table S1 and Fig. 2). PCR fragments targeting one sequence exhibited length polymorphism between the restored plants and the non-restored plants [a DNA fragment length polymorphism (DFLP) marker] (prefixed by ‘df’ in Table S1 and Fig. 2). The remaining five sequences were simultaneously amplified from both restored and non-restored plants. The reason why this occurred may be that the original AFLP between restored and non-restored bulks was generated because of alteration(s) within or close to the *Eco*RI- and/or the *Mse*I restriction endonuclease sites but the nucleotide sequence of the internal AFLP fragment was preserved. Concerning these five sequences, nucleotide sequences of PCR fragments amplified from a restored plant and a ‘TA-33BB-CMS’ plant were compared to find the sequence alterations in any of the restriction endonuclease recognition site, and we confirmed polymorphisms in the restriction patterns of the PCR fragments (CAPS markers) (prefixed by ‘ca’ in Table S1 and Fig. 2). The polymorphisms of these 17 STS markers exactly matched with those of their cognate AFLP fragments in the 25 BC_1_F_1_ plants that were used for BSA.

### *Rf2* is located on chromosome IV

We next tested whether the 17 STS markers were linked to each other. Segregation of each of the 17 STS markers in 115 BC_1_F_1_ plants statistically fits with the expected genetic model (Table S2). These segregation data were analyzed using mapping software. The resultant linkage map contained all the 17 STS markers, covering 28.1 cM (Fig. 2a). Chromosomal assignment of this linkage map was investigated using DNA markers developed by Schneider et al. (1999). Chromosome IV, which contains *Z*, was the most likely candidate; therefore, we tested the linkage of two chromosome-IV-assigned markers, *nir* and *ant*, to our map. As a result, *nir* and *ant* were found to flank our linkage map (Fig. 2a). Consequently the linkage map was expanded to 46.1 cM.

We examined whether *Rf2* could be mapped on our linkage map. Under the dichotomic assumption (i.e., restored vs. non-restored), we failed to map *Rf2* on our linkage map. As such, we next assumed that the observed fertility restoration was the quantitative trait involving *Rf2*. The map position of *Rf2* was analyzed by QTL analysis using male-fertility values. In the linkage map shown in Fig. 2a, both SIM and CIM methods detected a QTL for fertility restoration in the region between sc4 and a locus containing ca2, sc3, sc7 and sc10 (LOD = 36.15 and 27.68 for SIM and CIM, respectively) (Table 4). The confidence intervals (CIs) identified by SIM and CIM were within the map position of 13.6–15.3 cM in Fig. 2a, the region delimited by sc4 and ca2 (representing four markers). The two methods detected no other consistent QTL for fertility restoration.

**Table 4:** Summary of the QTL analysis

Population	Total number of plants	QTL for fertility restoration							
		SIM				CIM			
		Confidence interval^a^	LOD	*R* ^2^	Additive	Confidence interval^a^	LOD	*R* ^2^	Additive
BC1F1	115	13.6–15.3	36.15	0.77	1.97	13.6–15.3	27.68	0.51	1.83
BC1F3	184	12.8–16.8	24.93	0.81	0.89	12.0–17.0	32.7	0.59	0.97

^a^Map position in Fig. 2

We then examined the segregation of the 17 DNA markers, *nir*, and *ant* in the BC_1_F_3_ (Table S3). Two markers, sc1 and ca4, could not be mapped because all the plants were homozygous for ‘TA-33BB-CMS’-type alleles. Segregation of the other markers fits with the expected genetic model (Table S3), and we analyzed these data using the mapping software. As a result, we obtained a map of 34.5 cM (Fig. 2b). The arrangement of markers is fairly well preserved between the BC_1_F_1_ and the BC_1_F_3_ (Fig. 2).

We conducted QTL analysis for fertility restoration in the BC_1_F_3_ to map *Rf2*. The highest LOD peak for fertility restoration was in the region delimited by sc11 and a locus containing sc3 and four other DNA markers, in which map positions of CI were 12.8–16.8 for SIM (LOD = 24.93) and 12.0–17.0 for CIM (LOD = 32.7) (Table 4; Fig. 2b). No other QTLs for fertility restoration were consistently detected by the two methods. The map position of the QTL for fertility restoration was very similar between the BC_1_F_1_ and BC_1_F_3_ and both of the detected QTLs associated with sc3, sc4, sc7, sc10, and ca2.

The closest markers to *Rf2* appeared to be sc4, sc3, sc7, sc10 and ca2, because the presence or absence of these five markers showed the best association with male-fertility restoration (107/114 in the BC_1_F_1_ and 132/146 in the BC_1_F_3_). Therefore, *Rf2* was located in the interval between sc4 and ca2 (one of four markers, see Fig. 2a), ~13.6 to ~15.3 cM away from *nir* toward *ant* (BC_1_F_1_), or located near the site containing the five markers, ~8.8 cM away from *nir* toward *ant* (BC_1_F_3_).

## Discussion

Fertility restoration in Owen-CMS is a very complex trait, as pointed out by previous investigations, in which various segregation patterns were described (Owen 1945; Hogaboam 1957; Bliss and Gabelman 1965; Nagao and Kinoshita 1962; Theurer and Ryser 1969; Hjerdin-Panagopoulos et al. 2002). This complexity likely came from the combined action of major and minor *Rf*s, as well as environmental factors (Owen 1945). Hence, genetic dissection of this phenotype is a prerequisite to assessing the action of each *Rf*, a necessary procedure for genetic mapping. Before this study, the major role of *Rf1* in fertility restoration was proposed (e.g. Moritani et al. 2013). We intended to eliminate the action of *Rf1* for the accurate genetic analysis of the minor *Rf*.

Although *Rf2* is a minor *Rf* in terms of genetics, the significance of *Rf2* on sugar beet breeding is another issue. In other words, if *Rf2* is practically important, this locus cannot be ignored by breeders, irrespective of its strength. In the case of *Rf1*, Japanese breeders have been carefully eliminating the restoring *Rf1* allele during maintainer selection, resulting in the selection of a few non-restoring alleles from varieties of *Rf1* alleles (Moritani et al. 2013). This notion was supported by the analysis of the allelic frequency of *Rf1* in the ancestral populations of Japanese sugar beet (Taguchi et al. 2014), suggesting a major impact of *Rf1* on maintainer selection. We speculate that *Rf2* may be another target locus for maintainer-genotype selection for the following reason: although the effect of *Rf2* on fertility restoration is small, partially fertile plants in the test-cross progeny will be easily recognized by breeders as a sign of a non-maintainer genotype. In fact, partial male fertility of test-cross progeny derived from ‘TA-36’ was sufficient to make Moritani et al. (2013) reject all the *rf1rf1* plants as candidates of the maintainer. The question of whether *Rf2* has been one of the target loci for maintainer selection can be tested by examining the molecular polymorphism of *Rf2* in sugar beet lines. Currently, we are working to identify the nucleotide sequence of *Rf2*.

The effect of *Rf2* is obviously weak because no fully fertile plants were obtained in this study and the plants are at the most partially fertile. As seen in the male-fertility values, fertility restoration in the BC_1_F_1_ and the BC_1_F_3_ appeared to be a quantitative trait. Considering that the BC_1_F_1_- (and the BC_1_F_3_-) fertility restoration differed within a plant, it is possible that the penetrance of *Rf2* is influenced by the plant’s physiological condition (nutrition, age, etc.). In addition, it is possible that other minor *Rf*(s) and/or environmental factors may be involved. Thus, one of the remaining questions is how the phenotypic expression of *Rf2* changes in relation to other factors. This is an important question, because if the action of *Rf2* can be masked for some reason, the *Rf2* allele might contaminate CMS lines through maintainer lines, potentially decreasing hybrid purity.

*Rf2* is situated in the region delimited by *nir* and *ant* on chromosome IV, on which *Z* is the only *Rf* known to be located (Schondelmaier and Jung 1997). Thus *Rf2* is likely an allele of *Z*. In addition, the low restoration effect of *Rf2* is consistent with the postulation of *Z* provided by many sugar beet researchers (Owen 1945; Hogaboam 1957; Theurer 1971).

In this context, we think *Rf2* may correspond to one of the two-linked QTLs for fertility restoration reported by Hjerdin-Panagopoulos et al. (2002). The quantitative nature of *Rf2* is consistent with their result. The gene corresponding to the other QTL (i.e., the third *Rf*) was not detected in our study, possibly because the third *Rf* is absent from ‘E60’. It is not surprising that the composition of minor *Rf*s differs between sugar beet lines, considering the various segregation patterns of fertility restoration in the previous investigations (see above for references). Unfortunately, because no DNA marker in this study is shared with those of Hjerdin-Panagopoulos et al. (2002), further comparison of the results is impossible. However, the map position of *Rf2* and the markers developed in this study would be useful to compare sugar beet *Rf*s between different populations. The overarching genetic and environmental control of fertility restoration of Owen-CMS should be investigated for the benefit of sugar beet breeding.

### **Author contributions**

KT, TM and TK designed this study; KT developed all the plant materials; YH, KT, HH and RYK performed the experiments; YH, KT and TK analyzed the data; YH, KT and TK wrote the manuscript.

## Electronic supplementary material

Below is the link to the electronic supplementary material.

Supplementary material 1 (PDF 34 kb)

Supplementary material 2 (XLS 51 kb)

Supplementary material 3 (XLS 75 kb)
