# Supplementary material for: Molecular mapping of restorer-of-fertility 2 gene identified from a sugar beet (Beta vulgaris L. ssp. vulgaris) homozygous for the non-restoring restorer-of-fertility 1 allele
Source: Theor Appl Genet. 2014 Oct 7;127(12):2567–74. doi: 10.1007/s00122-014-2398-4 (PMC4236623; doi:10.1007/s00122-014-2398-4)
Supplement: Supplementary file 1 — Supplementary material 1 (PDF 34 kb) [file 122_2014_2398_MOESM1_ESM.pdf]

Table S1 DNA markers and the nucleotide sequences of primers used in this study

Name of markers	Size (bp)	Forward primer (5'--3')	Reverse primer (5'--3')	Marker type (Restriction endonuclease)	Source
sc1	319	AATACAATCCCTCAAGTATGTCGA	TTAGCAACATGTCAGTGCTTGATG	SCAR	This study
sc2	524	AAGTATTTGACTAAGGCTGGTAG	GCTTAGGGGGTAAGTGTCATA	SCAR	This study
sc3	788	AAGTCGATCTCCTCCACCTT	GGATGTACTGAGTATGAGAGAATA	SCAR	This study
sc4	243	CGCTTGTCTCAATTCACATCA	TTGAGACCTCTATTTCTGCAG	SCAR	This study
sc5	382	GATTGCCCAGGGTTCGAA	GTCCCACGTAGCAGACAT	SCAR	This study
sc6	462	GTTTGGCGGTGAACAGGAC	AGAAACTGGGCTCCAGCAG	SCAR	This study
sc7	377	GTAGTGTGCATATGTATCTCGT	TGAGGAGTGATTGAAGGTGAGT	SCAR	This study
sc8	333	CAGGATGCCACCGTTCTCAAG	TCGTGATACCTCCTCGGTCA	SCAR	This study
sc9	169	GTCCATCTCATTACTTTGGGA	TTTCCAACTCAATAACAAATTCAG	SCAR	This study
sc10	435	GCCTGACCAGCCCAGAAGGCA	TCGGCTCAATGCGGGATCC	SCAR	This study
sc11	319	TCTGCACCTAATTTTTTTCATGCC	AAGATCAAAGCTCTACTCCATAAC	SCAR	This study
df1	390	GAGCGCCAACTCCAATGTC	GCTTTCTTCAGTGTTAGTGACTC	DFLP	This study
ca1	313	TGTTATACCTATGCCACTTGAAG	TCAAGTTCGAACATTGGTGTCGA	CAPS (*Alu*I)	This study
ca2	228	CTTCGCATTCCATCATAATGTTTG	CTCACAAGTCATAACCGCGT	CAPS (*Eco*RI)	This study
ca3	323	AGAGCGTCGTCTTTCTCGG	TCTCTCTCCTCTAATCAGACACT	CAPS (*Taq*I)	This study
ca4	612	GAGAACATGAAATTGCTGCCTG	GTCCACCGGAAGAATGACC	CAPS (*Hin*dIII)	This study
ca5	222	AACCAGAGCATATCTCTCTAGC	GGCATCGGGTCAAATCTCAC	CAPS (*Mbo*I)	This study
nir	1600	GTTAGRCTCAAGTGGCTTGG	GGCATTCTCTTCTCWACCTC	CAPS (*Rsa*I)	Schneider et al. 1999
ant	1000	TGGAGAGGAAACACTGCYAATGT	ATGTTRGCACCAGCWCCCTTGA	CAPS (*Taq*I)	Schneider et al. 1999
